# Reduced Heart Rate Recovery Is Associated with Poorer Cognitive Function in Older Adults with Cardiovascular Disease

**DOI:** 10.1155/2012/392490

**Published:** 2012-09-04

**Authors:** Therese A. Keary, Rachel Galioto, Joel Hughes, Donna Waechter, Mary Beth Spitznagel, James Rosneck, Richard Josephson, John Gunstad

**Affiliations:** ^1^Department of Psychology, Kent State University, Kent, OH 44242, USA; ^2^Center for Cardiopulmonary Researsh, Summa Health System, Akron, OH 44398, USA; ^3^University Hospitals Case Medical Center and Department of Medicine, Case Western Reserve University, Cleveland, OH 44106, USA; ^4^Harrington Heart & Vascular Institute, Case Western Reserve University, Cleveland, OH 44106, USA; ^5^School of Medicine, Case Western Reserve University, Cleveland, OH 44106, USA

## Abstract

Cardiovascular disease (CVD) in older adults has been associated with varying degrees of cognitive dysfunction. Several mechanisms may explain this association, including impaired cardiovascular reactivity to autonomic nervous system (ANS) signaling. Reduced heart rate recovery following a stress test may be considered an indication of impaired ANS function (i.e., reduced parasympathetic activity). Participants were 47 older adults (53–83 years) who underwent a treadmill stress test and were administered a comprehensive neuropsychological battery upon entry to phase II cardiac rehabilitation. Reduced parasympathetic activity was associated with impaired cognitive performance on a measure of global cognitive function and on tasks of speeded executive function and confrontation naming. These relationships suggest that changes in autonomic function may be mechanistically related to the impaired cognitive function prevalent in CVD patients.

## 1. Introduction

Cardiovascular disease (CVD) is associated with varying degrees of cognitive impairment, ranging from minimal difficulties (“brain at risk stage”) to dementia [1–4] and is associated with cognitive decline above and beyond the normal aging process [5]. Impaired cognitive function is observed in persons with CVD, even in the absence of major cardiac events [6]. Older adults with CVD frequently report experiencing significant cognitive dysfunction in everyday life and men with CVD demonstrate a reduction in cognitive function equivalent to approximately four to five years of additional age [7, 8]. These findings are in addition to the known associated between CVD and severe neurological conditions like stroke and Alzheimer's disease [9, 10]. 

Several pathophysiological mechanisms associated with CVD contribute to the observed cognitive dysfunction in this population. For example, systemic hypoperfusion is common and associated with reduced cognitive test performance [11–13]. Similarly, CVD is associated with development of white matter disease [14], pathological changes to blood vessels [15], and inflammatory processes [16], each of which are known to adversely impact cognitive function. Recent work implicates disrupted autonomic nervous system (ANS) signaling as another potential mechanism for the cognitive impairment, as both blood pressure variability (e.g., standard deviation of systolic blood pressure) and heart rate variability have been linked to neurocognitive outcome in persons with CVD [17–19].

 Such findings suggest that other, more easily obtained ANS indices may also be associated with reduced cognitive function and provide insight into potential mechanisms. One such ANS index is heart rate recovery, which may be operationally defined as the change from peak HR following maximal exercise (typically during treadmill stress testing) to that measured after two minutes of recovery [18]. This commonly used ANS index is sensitive to parasympathetic activity, as the return to resting HR following exercise termination is predominately accomplished via vagus nerve activity [19–21]. 

To help clarify the association between HR recovery and cognitive function, we assessed a sample of both men and women (age range 53–83 years) with a wide range of CVD conditions using a comprehensive neuropsychological test battery and a treadmill stress test. We hypothesized that ANS dysfunction would be associated with poorer cognitive function, such that greater HR recovery would correspond to better cognitive function. 

## 2. Methods 

### 2.1. Participants

The study sample included 47 (32 men, 15 female) older adults enrolled in a prospective study of the neurocognitive consequences of CVD. All participants were referred by their cardiologists for enrollment into a large cardiac rehabilitation program; information regarding the type of CVD event or condition diagnosis which prompted referral to cardiac rehabilitation can be found in Table 1. The mean time interval between the CVD event or condition diagnosis and the stress test is 36.3 days (minimum = 8, maximum = 147; median = 34). Participants in the study sample had a wide range of CVD conditions, including arrhythmia, atrial fibrillation, coronary artery bypass graft (CABG), valve surgery, cardiac arrest, myocardial infarction, heart failure, and hypertension; see Table 1. 

The exclusion criteria in the current student included (1) current signs of dementia (as defined by a score lower than 24 on the Mini Mental Status Exam (MMSE)) [23], (2) history of a major neurological disorder such as Alzheimer's disease, Parkinson's disease, seizures, large vessel stroke, or loss of consciousness >10 minutes, and (3) history of major psychiatric disorder such as schizophrenia, bipolar illness, or substance abuse. In addition, no participants with new onset or chronic atrial arrhythmia were included in the study sample. Participants were not selected based on concerns regarding possible cognitive dysfunction. Participants averaged 67.87 ± 8.92 years of age and 14.07 ± 2.61 years of education. All participants were Caucasian. 

On the basis of medical chart review, clinically relevant conditions (i.e., arrhythmia, atrial fibrillation, coronary artery bypass graft (CABG), cardiac arrest, type II diabetes, myocardial infarction, heart failure, high cholesterol, hypertension, and *β*-blocker usage) were coded as either present or absent; see Table 1.

### 2.2. Measures

#### 2.2.1. Exercise Stress Testing

 Resting HR, peak HR, exercise capacity, and HR recovery values for each participant were obtained using a treadmill exercise test. The treadmill exercise test was conducted according to a modified ramp protocol using a Quinton Medtrack ST55 treadmill (Quinton Cardiology, Deerfield, WI). More specifically, the exercise protocol was characterized by an increase in mill elevation every 60 seconds that approximates a relative increase in workload of 15% holding speed constant, as well as an increase in speed every 3 minutes that also reflects an approximate relative increase in workload of 15% from the previous stage. The test was terminated if the patient showed clinical signs of cardiovascular, neurological, or musculoskeletal decompensation, or at the patient's request; such practice is consistent with American College of Cardiology/American Heart Association and American College of Sports Medicine published guidelines for exercise stress testing [24–26]. Exercise capacity was estimated in metabolic equivalents (METS) at this peak volition or symptom limited end point [24]. 

In the present study, HR recovery was operationally defined as the difference in beats per minute (bpm) between peak HR (i.e., HR at exercise termination) and HR two minutes after exercise termination [20, 21, 27]. Although HR recovery has also been computed from HR one minute after exercise termination, HR recovery at 2 minutes predicted mortality better than other indices in a large study of male patients completing treadmill tests and coronary angiography [28], and, in another study, only HR recovery at 2 minutes predicted the presence of coronary artery disease [29]. In a validation study including 2,193 men, the average drop in HR at 2 minutes was 31.7 ± 13.1, and a reduction in HR of less than 22 bpm within 2 minutes following peak exercise was considered “abnormal” [28]. 

#### 2.2.2. Neuropsychological Tests

All participants completed a neuropsychological test battery comprised of tasks commonly used during clinical neuropsychological evaluation; see Table 1. The following four domains were assessed.

1. Global cognitive functioning using the Modified Mini Mental Status (3MS) [30].
2. Attention/executive function/psychomotor speed using the Trail Making Test A and B (TMT-A and TMT-B) [31], Letter-Number Sequencing (LNS) [32], Frontal Assessment Battery (FAB) [33], and Grooved Pegboard (Pegs) [34] dominant hand.
3. Memory using the Hopkins Verbal Learning Test (HVLT)—revised [35].
4. Language using the Boston Naming Test (BNT)—short form [22] and Animal Naming (animals) [36].

### 2.3. Procedure

All procedures were approved by the Institutional Review Board of the associated university prior to study onset. All participants provided written informed consent prior to study participation. Exercise stress testing was completed upon entry into cardiac rehabilitation. All neuropsychological instruments were individually administered by trained research team members. Neuropsychological testing was completed within 14 days of the exercise stress test. 

### 2.4. Data Analysis

In preliminary analyses, descriptive data regarding participant demographic characteristics, clinical conditions, cardiovascular indices, and neuropsychological test performance were generated. The primary analyses consisted of a series of partial correlations between HR recovery and the neuropsychological test performance variables. Control variables included those factors with known importance for HRR and/or cognitive function in older adults with CVD, specifically sex, age, years of education, *β*-blocker usage, and exercise capacity (as measured by METS at peak HR). Finally, in order to evaluate the relative strength of the findings, effect size values (i.e., Cohen's *d*) were calculated for all partial correlations. 

Previous research has documented a relationship depression and HR recovery in a very similar sample of phase II cardiac rehabilitation patients [21]. All participants in the present study completed the Beck Depression Inventory (BDI) [37] as part of the cardiac rehabilitation program. However, BDI total score was not significantly correlated with any of the neuropsychological test performance variables. In the absence of such correlation, the primary analyses were not adjusted for BDI scores.

## 3. Results

Participant demographic characteristics, clinical conditions, cardiovascular indices, reasons for referral to cardiac rehabilitation, and neuropsychological test performance are presented in Table 1. Of note, HR recovery values ranged from 0 bpm to 67 bpm (M = 31.7, SD = 15.2). 

Partial correlations and Cohen's *d* effect sizes between HR recovery and the neuropsychological test performance variables are presented in Table 2. After adjusting for relevant variables (i.e., sex, age, years of education, *β*-blocker usage, and exercise capacity), HR recovery was significantly correlated with 3MS total score (*r* = .42, *P* = .006), BNT short-form total score (*r* = .40, *P* = .008), and TMT-B time to completion (*r* = −.33, *P* = .03). Associations for 3MS and BNT short form indicate large effect sizes (Cohen's *d* values of  .93 and  .87, resp.), while the association for TMT-B indicates a medium effect (Cohen's *d* = .70). The correlations between HR recovery and both TMT-A time to completion (*r* = −.28, *P* = .08) and grooved pegboard time to completion (*r* = −.28, *P* = .08) approached statistical significance and were consistent with a medium effect size (Cohen's *d* = .58 for both measures).

## 4. Discussion

The results of the present study indicate that greater heart rate recovery two minutes after exercise termination is associated with better performance in multiple cognitive domains, including global cognitive function, speeded executive function, and language. Several aspects of these findings warrant brief discussion.

The current findings extend previous research [38] and demonstrate that the relationships between ANS-mediated cardiovascular functioning and cognitive tasks are independent of cardiovascular fitness (as measured by exercise capacity). Although the specific mechanisms underlying these associations are currently unknown, we offer several candidate explanations. One likely explanation involves the effects of ANS dysregulation on vascular processes. Endothelial dysfunction and impaired cardiovascular reactivity to ANS signaling are common in CVD patients [39–44] and have been suggested as a contributor to cognitive dysfunction in other studies [45]. An additional explanation implicates the inflammatory cytokines of the vagus nerve; more specifically, both slowed HR recovery and cognitive dysfunction may be a reflection of an underlying dysregulation of the cholinergic anti-inflammatory pathway [46]. Another possibility is that poorer HR recovery and cognitive function reflect structural brain changes such as white matter disease or reduced integrity of white matter pathways. For example, the anterior cingulate cortex is important for both ANS functioning and frontal lobe-mediated functions (e.g., attention/executive function/psychomotor speed) [47, 48] and damage in this region could well affect both indices. Future neuroimaging studies utilizing standard imaging and diffusion tensor imaging technology [49] will help to clarify this possibility.

Identifying a relationship between a measure of cardiac function and measures of global cognitive function and speeded executive function is consistent with past studies [50, 51]. However, finding an association between HR recovery and language function is less common. One possibility is that HR recovery is an underlying marker for neurodegenerative conditions with known language impairment, such as Alzheimer's disease. CVD has long been known as a risk factor for Alzheimer's disease [52, 53]. Persons with Alzheimer's disease exhibiting autonomic cardiac dysfunction [54], manifesting as a relatively hypersympathetic and hypo-parasympathetic state [55]. Thus, it may be possible that indices of ANS function (e.g., HR recovery, HR variability) may prove to be proximal predictors of Alzheimer's disease [56]. 

The current findings are limited in several ways. First, due to the cross-sectional design of the present study, it is not clear whether the association between HR recovery and cognitive function simply represents a cooccurrence or reflects a casual relationship. Future studies with a longitudinal design may clarify this issue. Longitudinal investigations may also examine whether improvements in HR recovery correspond with the cognitive improvements found in CVD patients in cardiac rehabilitation [57–59]. Second, the present study examined cognitive functioning within a diverse group of CVD conditions and did not attempt to make any differentiation among them. Although the medium to large effect sizes suggest the findings are robust, replication and extension of the current findings in larger samples with more narrowly defined CVD patient samples are needed. Similarly, future studies should also examine the association between HR recovery and cognitive function in older adults without CVD to help clarify possible mechanisms for this pattern. 

In brief summary, the current study extends past work to show that HR recovery following a treadmill stress test (an index of autonomic nervous system function) is related to multiple cognitive domains, including global cognitive functioning, speeded executive function, and language abilities. Further work is needed to clarify possible mechanisms for these findings and to explore potential for therapeutic improvement.

## Figures and Tables

**Table 1:** Demographic characteristics, clinical conditions, reasons for referral to cardiac rehabilitation, cardiovascular indices, and neuropsychological test performance (*N* = 47).

Demographics	Mean (SD)	Range
Age (years)	67.87 (8.92)	53–83
Education (years)	14.07 (2.61)	7–20
Male, *N*(%)	32 (68%)	15

Clinical conditions	Percentage with condition	

Arrhythmia	2%	
Atrial fibrillation	11%	
Coronary artery bypass graft (CABG)	23%	
Cardiac arrest	2%	
Diabetes (type II)	26%	
Myocardial infarction (MI)	21%	
Heart failure	15%	
High cholesterol	28%	
Hypertension	43%	
*β*-blocker usage	68%	

Type of event or condition prompting referral to cardiac rehabilitation	Number of participants	Percentage of participants

Ablation	1	2%
Angina	5	11%
Aortic valve repair	1	2%
CABG	8	17%
MI	6	13%
Mitral valve repair	3	6%
Stent	21	45%
Valve replacement	2	4%

Cardiovascular indices	Mean (SD)	Range; median

HR_rest_*	66.6 (11.9)	40–96; 65
HR_peak_	112.7 (19.5)	60–159; 115
METs_peak_	7.3 (3.2)	1.0–14.5; 7.0
HR_2min-post_	81.0 (18.2)	43–126; 80
Heart rate recovery	31.7 (15.2)	0–67; 31

Neuropsychological test performance	Mean (SD)	Range

MMSE	28.4 (1.5)	25–30
3MS	95 (4.1)	83–100
TMT-A time (seconds)	36.9 (11.7)	22–82
TMT-B time (seconds)	97.3 (49.9)	42–325
LNS total score	8.9 (3.00)	2–15
FAB total score	16.4 (1.7)	11–18
Pegs, dominant hand time (seconds)	103.4 (52.5)	56–398
HVLT learning	21.9 (5.5)	10–33
HVLT delayed recall	7.2 (3.4)	0–12
HVLT true hits	11.0 (1.2)	7–12
BNT short form total score	14.3 (0.9)	12–15
Animal Naming	18.3 (5.2)	7–33

*Data regarding resting HR (HR_rest_) were missing for 1 of the 47 participants.

**Table 2:** Correlations between heart rate recovery and neuropsychological test performance, controlling for sex, age, years of education, *β*-blocker usage, and METS at peak HR (*N* = 47).

	Heart rate recovery	
	Partial correlation *r* (*P* value)	Cohen's *d* effect size
3MS	.42 (.006)**	.93^c^
TMT-A time (seconds)	−.28 (.07)	.58^b^
TMT-B time (seconds)	−.33 (.03)*	.70^b^
LNS total score	.17 (.29)	.35^a^
FAB total score	.12 (.44)	.24^a^
Pegs, dominant hand time (seconds)	−.28 (.08)	.58^b^
HVLT learning	.19 (.23)	.39^a^
HVLT delayed recall	.15 (.35)	.30^a^
HVLT true hits	.25 (.11)	.52^b^
BNT—short form total score	.40 (.008)**	.87^c^
Animal naming	.13 (.41)	.26^a^

*P <* *.05, **P <* *.01; Cohen's *d* values of 0.2, 0.5, and 0.8 are the minimum thresholds for a small effect^a^, medium effect^b^, and large effect^c^ sizes, respectively [22].

## References

[1] Desmond DW (2004). The neuropsychology of vascular cognitive impairment: is there a specific cognitive deficit?. *Journal of the Neurological Sciences*.

[2] Rafnsson SB, Deary IJ, Smith FB, Whiteman MC, Fowkes FGR (2007). Cardiovascular diseases and decline in cognitive function in an elderly community population: the Edinburgh Artery study. *Psychosomatic Medicine*.

[3] Trojano L, Incalzi RA, Acanfora D, Picone C, Mecocci P, Rengo F (2003). Cognitive impairment: a key feature of congestive heart failure in the elderly. *Journal of Neurology*.

[4] Hachinski V (1994). Vascular dementia: a radical redefinition. *Dementia*.

[5] Okonkwo OC, Cohen RA, Gunstad J, Tremont G, Alosco ML, Poppas A (2010). Longitudinal trajectories of cognitive decline among older adults with cardiovascular disease. *Cerebrovascular Diseases*.

[6] Duron E, Hanon O (2008). Vascular risk factors, cognitve decline, and dementia. *Vascular Health and Risk Management*.

[7] Elwood PC, Pickering J, Bayer A, Gallacher JEJ (2002). Vascular disease and cognitive function in older men in the Caerphilly cohort. *Age and Ageing*.

[8] Khatri P, Babyak M, Clancy C (1999). Perception of cognitive function in older adults following coronary artery bypass surgery. *Health Psychology*.

[9] Kivipelto M, Helkala EL, Laakso MP (2001). Midlife vascular risk factors and Alzheimer’s disease in later life: longitudinal, population based study. *British Medical Journal*.

[10] Witt BJ, Ballman KV, Brown RD, Meverden RA, Jacobsen SJ, Roger VL (2006). The incidence of stroke after myocardial infarction: a meta-analysis. *The American Journal of Medicine*.

[11] Jefferson AL, Poppas A, Paul RH, Cohen RA (2007). Systemic hypoperfusion is associated with executive dysfunction in geriatric cardiac patients. *Neurobiology of Aging*.

[12] Román GC (2004). Brain hypoperfusion: a critical factor in vascular dementia. *Neurological Research*.

[13] Román GC (2005). Vascular dementia prevention: a risk factor analysis. *Cerebrovascular Diseases*.

[14] Longstreth WT, Arnold AM, Beauchamp NJ (2005). Incidence, manifestations, and predictors of worsening white matter on serial cranial magnetic resonance imaging in the elderly: the cardiovascular health study. *Stroke*.

[15] Moser DJ, Hoth KF, Robinson RG (2004). Blood vessel function and cognition in elderly patients with atherosclerosis. *Stroke*.

[16] Gunstad J, Bausserman L, Paul RH (2006). C-reactive protein, but not homocysteine, is related to cognitive dysfunction in older adults with cardiovascular disease. *Journal of Clinical Neuroscience*.

[17] Okonkwo OC, Cohen RA, Gunstad J, Poppas A (2011). Cardiac output, blood pressure variability, and cognitive decline in geriatric cardiac patients. *Journal of Cardiopulmonary Rehabilitation and Prevention*.

[18] Shah AJ, Su S, Veledar E (2011). Is heart rate variability related to memory performance in middle-aged men?. *Psychosomatic Medicine*.

[19] Thayer JF, Hansen AL, Saus-Rose E, Johnsen BH (2009). Heart rate variability, prefrontal neural function, and cognitive performance: the neurovisceral integration perspective on self-regulation, adaptation, and health. *Annals of Behavioral Medicine*.

[20] Cole CR, Foody JM, Blackstone EH, Lauer MS (2000). Heart rate recovery after submaximal exercise testing as a predictor of mortality in a cardiovascularly healthy cohort. *Annals of Internal Medicine*.

[21] Hughes JW, Casey E, Luyster F (2006). Depression symptoms predict heart rate recovery after treadmill stress testing. *American Heart Journal*.

[22] Kaplan E, Goodglass H, Weintraub S (1983). *The Boston Naming Test*.

[23] Folstein MF, Folstein SE, McHugh PR (1975). ‘Mini mental state’: a practical method for grading the cognitive state of patients for the clinician. *Journal of Psychiatric Research*.

[24] American College of Sports Medicine (2000). *ACSM's Guidelines for Exercise Testing and Prescription*.

[25] Fletcher GF, Balady GJ, Amsterdam EA (2001). Exercise standards for testing and training: a statement for healthcare professionals from the American Heart Association. *Circulation*.

[26] Gibbons RJ, Balady GJ, Bricker JT (2002). ACC/AHA 2002 guideline update for exercise testing: summary article. A report of the American College of Cardiology/American Heart Association task force on practice guidelines. *Journal of the American College of Cardiology*.

[27] Lipinski MJ, Vetrovec GW, Froelicher VF (2004). Importance of the first two minutes of heart rate recovery after exercise treadmill testing in predicting mortality and the presence of coronary artery disease in men. *American Journal of Cardiology*.

[28] Shetler K, Marcus R, Froelicher VF (2001). Heart rate recovery: validation and methodologic issues. *Journal of the American College of Cardiology*.

[29] Lipinski MJ, Vetrovec GW, Froelicher VF (2004). Importance of the first two minutes of heart rate recovery after exercise treadmill testing in predicting mortality and the presence of coronary artery disease in men. *American Journal of Cardiology*.

[30] Teng EL, Chui HC (1987). The Modified Mini-Mental State (3MS) examination. *Journal of Clinical Psychiatry*.

[31] Reitan RM, Wolfson D (1993). *The Halstead-Reitan Neuropsychological Test Battery: Theory and Clinical Interpretation*.

[32] Wechsler D (1997). *Wechsler Adult Intelligence Scale*.

[33] Dubois B, Slachevsky A, Litvan I, Pillon B (2000). The FAB: a frontal assessment battery at bedside. *Neurology*.

[34] Kløve H, Forster FM (1963). Clinical neuropsychology. *The Medical Clinics of North America*.

[35] Brandt J (1991). The Hopkins Verbal Learning Test: development of a new memory test with six equivalent forms. *Clinical Neuropsychologist*.

[36] Eslinger P, Damasio AR, Benton AL (1984). *The Iowa Screening Battery For Mental Decline*.

[37] Beck AT, Rush AJ, Shaw BF, Emery G (1979). *Cognitive Therapy of Depression*.

[38] Hansen AL, Johnsen BH, Sollers JJ, Stenvik K, Thayer JF (2004). Heart rate variability and its relation to prefrontal cognitive function: the effects of training and detraining. *European Journal of Applied Physiology*.

[39] Carney RM, Freedland KE, Veith RC (2005). Depression, the autonomic nervous system, and coronary heart disease. *Psychosomatic Medicine*.

[40] Dekker JM, Crow RS, Folsom AR (2000). Low heart rate variability in a 2-minute rhythm strip predicts risk of coronary heart disease and mortality from several causes: the ARIC study. *Circulation*.

[41] Harris KF, Matthews KA (2004). Interactions between autonomic nervous system activity and endothelial function: a model for the development of cardiovascular disease. *Psychosomatic Medicine*.

[42] Hassan A, Hunt BJ, O’Sullivan M (2004). Homocysteine is a risk factor for cerebral small vessel disease, acting via endothelial dysfunction. *Brain*.

[43] Hijmering ML, Stroes ESG, Olijhoek J, Hutten BA, Blankestijn PJ, Rabelink TJ (2002). Sympathetic activation markedly reduces endothelium-dependent, flow-mediated vasodilation. *Journal of the American College of Cardiology*.

[44] Neki NS, Singh RB, Rastogi SS (2004). How brain influences neuro-cardiovascular dysfunction. *Journal of Association of Physicians of India*.

[45] Hoth KF, Tate DF, Poppas A (2007). Endothelial function and white matter hyperintensities in older adults with cardiovascular disease. *Stroke*.

[46] Tracey KJ (2007). Physiology and immunology of the cholinergic antiinflammatory pathway. *Journal of Clinical Investigation*.

[47] Critchley HD, Mathias CJ, Josephs O (2003). Human cingulate cortex and autonomic control: converging neuroimaging and clinical evidence. *Brain*.

[48] Matthews SC, Paulus MP, Simmons AN, Nelesen RA, Dimsdale JE (2004). Functional subdivisions within anterior cingulate cortex and their relationship to autonomic nervous system function. *NeuroImage*.

[49] DaSilva AF, Tuch DS, Wiegell MR, Hadjikhani N (2003). A primer on diffusion tensor imaging of anatomical substructures. *Neurosurg Focus*.

[50] Vicario A, Martinez CD, Baretto D, Diaz Casale A, Nicolosi L (2005). Hypertension and cognitive decline: impact on executive function. *Journal of Clinical Hypertension*.

[51] Vinkers DJ, Stek ML, van der Mast RC (2005). Generalized atherosclerosis, cognitive decline, and depressive symptoms in old age. *Neurology*.

[52] de La Torre JC (2006). How do heart disease and stroke become risk factors for Alzheimer’s disease?. *Neurological Research*.

[53] Newman AB, Fitzpatrick AL, Lopez O (2005). Dementia and Alzheimer’s disease incidence in relationship to cardiovascular disease in the Cardiovascular Health Study cohort. *Journal of the American Geriatrics Society*.

[54] Giubilei F, Strano S, Imbimbo BP (1998). Cardiac autonomic dysfunction in patients with Alzheimer disease: possible pathogenetic mechanisms. *Alzheimer Disease and Associated Disorders*.

[55] Aharon-Peretz J, Harel T, Revach M, Ben-Haim SA (1992). Increased sympathetic and decreased parasympathetic cardiac innervation in patients with Alzheimer’s disease. *Archives of Neurology*.

[56] Zulli R, Nicosia F, Borroni B (2005). QT dispersion and heart rate variability abnormalities in Alzheimer’s disease and in mild cognitive impairment. *Journal of the American Geriatrics Society*.

[57] Myers J, Hadley D, Oswald U (2007). Effects of exercise training on heart rate recovery in patients with chronic heart failure. *American Heart Journal*.

[58] Gunstad J, MacGregor KL, Paul RH (2005). Cardiac rehabilitation improves cognitive performance in older adults with cardiovascular disease. *Journal of Cardiopulmonary Rehabilitation*.

[59] Tiukinhoy S, Beohar N, Hsie M (2003). Improvement in heart rate recovery after cardiac rehabilitation. *Journal of Cardiopulmonary Rehabilitation*.

